# The relationship between a less gender-stereotypical parenthood and alcohol-related care and death: A registry study of Swedish mothers and fathers

**DOI:** 10.1186/1471-2458-8-312

**Published:** 2008-09-15

**Authors:** Anna Månsdotter, Mona Backhans, Johan Hallqvist

**Affiliations:** 1 Karolinska Institutet, Department of Public Health Science, Division of Social Medicine, SE-171 76 Stockholm, Sweden; 2 Swedish National Institute of Public Health, Research Department, SE-831 40 Östersund, Sweden

## Abstract

**Background:**

In general men tend to drink more alcohol and experience more alcohol-related sickness, injuries and mortality than women. In this paper, the overall hypothesis was that increased gender similarity in the division of parental duties would lead to convergence in alcohol-related harm. The aim was to analyse whether the risk of alcohol harm differs between parents who fit a gender-stereotypical versus those with a less gender-stereotypical division of childcare and paid work.

**Methods:**

The study sample was a retrospective registry-based cohort study of all Swedish couples who had their first child together in 1978 (N = 49,120). A less gender-stereotypical parenthood was indicated by paternity leave for fathers (1978–1979) and full-time work for mothers (1980). The outcome was inpatient care and/or death caused by alcohol psychosis, alcoholism, liver disease, or alcohol intoxication in the two decades following (1981–2001). Our main statistical method was multivariate logistic regression with odds ratios used to estimate relative risks.

**Results:**

The main results show that fathers who took paternity leave had 18% lower risk of alcohol-related care and/or death than other fathers. Mothers who worked full-time about two years after having a child had 71% higher risk than mothers who were unemployed or worked part-time.

**Conclusion:**

A less gender-stereotypical division of duties between parents in early parenthood may contribute to a long-term decreased gender disparity regarding risky alcohol consumption and alcohol-related harm. In order to know more about the causal direction however, future research has to consider subjects' drinking patterns in the years prior to parenthood.

## Background

All over the world, men drink more alcohol and have higher rates of alcohol-related health problems and mortality than women [1]. In fact the gender disparity in alcohol consumption is said to be one of few universal gender differences in human social behaviour [2]. The global difference may be illustrated by the percentage of disability-adjusted life years (DALYs) attributable to alcohol by sex: in high-mortality developing countries like Algeria, Bangladesh, and South Africa: males 2.6% and females 0.5%; in low-mortality developing countries like Argentina, Indonesia, and Turkey: males 9.8% and females 2.0%; and in low-mortality developed countries like USA, Japan, and Sweden: males 14.0% and females 3.3% [3].

The different males versus females drinking patterns are the result of both biological and social factors [4]. In this paper, the focus is on the gender system which refers to the societal structure organizing human activities and relations based on sex [5]. Fundamentally, fathers have historically been responsible for supporting the family (the 'breadwinner position'), while mothers have been responsible for taking care of the children and the home (the 'caring position') [6]. However, in the developed part of the world, many women have entered the public sphere of work, and some men have entered the private sphere of childcare. Our paper assesses alcohol-related consequences from a 'less gender-stereotypical division of parental duties' defined as a tendency among fathers to take on childcare duties and a tendency among mothers to take on 'breadwinner' obligations.

A basic theoretical concept in the paper is role theory, which generally explains human behaviour by expectations held by the individual as well as other people [7]. From this a number of concepts have emerged such as role confusion (dilemma in deciding which role to take on), role conflict (tension due to incompatible roles), and role embracement (hold a role so much that the self disappears). Two common theories regarding roles and health regards stress and expansion [8]. According to the *stress theory *[9] individuals with many activities and responsibilities experience more pressure, conflict and ill-health, because their primary role in life is so hard that additional duties risk health. The *expansion theory *contradicts this theory by suggesting that people with many roles have health advantages compared to others as they may compensate stress in one area with positive circumstances in other areas [10]. Most research on multiple roles and health has been based on women in the United States and Europe, and conclude that several life roles are mainly beneficial for lifetime health [11,12]. However, the relationship between the amount of roles and impact on health may also be curvilinear rather than linear; for example, a mother may begin to suffer from stress due to overwork instead of gaining from role expansion if the father does not take on his share of family and household duties [8,13,14].

One way of placing alcohol consumption in the context of role theory is that few roles (e.g. mother and housewife) may mean more time and opportunities to drink than if women have several roles (e.g. mother, housewife, friend, colleague, boss, and local politician). Moreover, gainful work for mothers may lead to lower rates of destructive behaviours including alcohol drinking through women having their own income, autonomy and social interaction [15], whilst childcare duties for fathers may translate to lower alcohol consumption through the satisfaction of adding closer relationships to their children [16]. Yet, the expansion of roles and responsibilities via extra public work (mothers) and extra private duties (fathers) beyond a certain level may also be so stressful that both sexes could seek for relaxation through alcohol consumption [8,15].

A further theoretical concept departs from the idea that a caring role may in itself be protective against risky lifestyles; the so called *caring theory *[16]. That is, one reason women drink less and live longer lives could be that they have had the main caring role and that alcohol poses a risk to the child's health and security; for example, duties such as picking up from day-care or discos are not compatible with regular and risky alcohol drinking [17]. Research shows that having dependent children implies a stronger protection for women than for men [18,19], but if fathers/men take on a substantive caring responsibility for their children, this may well apply to them as well [20].

Correspondingly, a weakened caring role for mothers/women could lead to a loss of health-protective incentives. That is, when females enter traditionally male spheres of life, they may experience traditionally male health risks, including risky alcohol consumption through more opportunities and less restrictions [21]. One reason that increased gender similarity in social positions has not implied gender similarity in lifestyles and alcohol drinking [22] is probably that women have retained the main caring (health-aware) position even after entering the paid labour market. Another explanation for findings indicating that women continue to drink less despite out-of-home employment may be that paid work has been measured by part-time work, which does not truly challenge the traditional division of the caring maternal position and the breadwinning paternal position [14].

Gender differences in a number of drinking measures have been examined by surveys in the general population in Europe (20–64 years, 2002) [23]. In Sweden, the setting of the present study, the male to female ratios were: 1.8 (abstainers), 1.5 (overall frequency of drinking), 1.5 (quantity in grams of pure alcohol), and 3.2 (frequency of episodic heavy drinking). In order to understand gender-specific drinking patterns one should also consider how socioeconomic position and aspects of the wider society, such as the welfare system and level of gender equality, interacts with gender [14,19,24]. A Swedish study has, for example, reported that 'high alcohol consumption' as well as 'intense drinking' is highest in single men with low education living in rural areas, while the similar female proportion is highest in well-educated women in non-manual occupations in urban areas [25]. Swedish alcohol consumption in around 1978, which was the historical start of our study period, was lower than it is today; 8.8 litres of 100% alcohol per inhabitant aged 15 years and over as compared to 10.4 litres in 2004 [26]. During the same period the male to female ratio in various drinking measures has been quite stable (around 2.0). Gender differences in drinking patterns and social interactions imply gender differences in sickness, injuries, and deaths; for example, 80% of the present mortality caused by alcoholic psychosis, alcoholism, cirrhosis of the liver, or alcohol poisoning in Sweden occurs among men [27].

The aim of the present study was to analyse whether the risk of alcohol-related inpatient care and/or death differs between parents who adopt a gender-stereotypical division of parental duties in early parenthood and those who adopt a *less *gender-stereotypical position as indicated by paternity leave for fathers and full-time work for mothers. The concepts of 'alcohol consumption' and 'drinking' will consider both frequency and quantity, while 'risky consumption/drinking' will refer to frequencies or quantities that are likely to imply alcohol-related inpatient care and/or death in the future.

Our overall hypothesis is that gender equality, in terms of more or less similarity between women and men in all spheres of life [28], leads to a convergence in of alcohol consumption and its consequences [14,29]. We anticipate that initial role expansion is associated with a decrease in risky drinking practices among both sexes, but also that the step from part-time to full-time work is linked with increased risky drinking in mothers. Besides, we acknowledge that childcare duties may per se protect against risky drinking. All in all, the theories of expansion, stress, and caring lead us to hypothesise a negative association between paternity leave and alcohol harm among men and a positive association between women's full-time work and alcohol harm. Finally, we recognise that the relationships could differ by age, occupational position, cohabitation status, whether the partner work full-time (in the male analysis), and whether the partner took parental leave (in the female analysis) [14,24], although these potential effect modifications were assessed from an explorative perspective.

## Methods

### Population

The study population was generated from the Multigenerational Register (Statistics Sweden) and comprises all Swedish couples (N = 49,120) who had their first child together in 1978. Based on the civil identification number assigned to all Swedish residents, personal information from different data sources were then linked to the parents.

The female study population in the regression analyses was from the outset restricted to 43,450 because of missing data on working hours (4,027) and exclusion of students (1,643). Additionally, as having another child two years after the birth of the first child (1978) is likely to influence men's decisions to take parental leave and women's decisions to work full-time, couples who had another child in 1979–1980 (n = 11, 089) were excluded from the regression analyses, which reduced the number of fathers to 38,031 and the number of mothers to 33,696 (i.e. 1,335 of the mothers who were students or missed data on working hours had another child in 1978–79). The regression analyses were restricted to include only those individuals with complete information on confounders which leaves 25,150 fathers and 33,406 mothers in the study populations.

### Outcome: alcohol-related care and/or death

The outcome variable was alcohol-related inpatient care and/or death (also referred to as 'alcohol harm') during the period 1981–2001, based on information attained from the Hospital Discharge Register and the Cause of Death Register (Swedish National Board of Health and Welfare). It was measured as inpatient care on at least one occasion and/or death, with alcohol psychosis, alcoholism, alcohol-related liver disease or alcohol intoxication as the discharge diagnosis or underlying or contributing cause of death. We did not include traffic accidents, drowning, suicides, and other consequences known to be alcohol-related as the registers do not state whether these are alcohol-related or not. The International Classification of Diseases codes used for both alcohol-related inpatient care and alcohol-related death were:

ICD-8 *(1981–1986)*: 291 (alcohol psychosis), 303 (alcoholism), 5710 (alcohol-related liver disease), E860, E980+N980 (alcohol intoxication)

ICD-9 *(1987–1996)*: 291 (alcohol psychosis), 303, 305.0, 357.5, 425.5, 535.3 (alcoholism including alcohol-related cardiovascular, stomach, and other disease), 571.0–571.3 (alcohol-related liver disease), E860, E980+980 (alcohol intoxication)

ICD-10 *(1997–2001)*: E24.4, F10.1–F10.9 (alcohol psychosis), G31.2, G62.1, G72.1, I42.6, K29.2, O35.4, Z71.4, Z50.2 (alcoholism including alcohol-related cardiovascular, stomach, and other disease, and treatment), K70, K86.0 (alcohol-related liver disease), T51, Y90.1–Y90.9, Y91.1–Y91.9 (alcohol intoxication)

The number of men with at least one alcohol-related diagnosis and/or alcohol-related death during the 21-year follow-up period was 2,196, of which: inpatient care – 1,874; death – 101; inpatient care and death – 221. The corresponding number for women was 899, of which: inpatient care – 810; death – 40; inpatient care and death – 49. The most common cause of death among males was consequences from alcoholism (30.6%) and among females alcohol intoxication (45.8%).

### Exposure: less gender-stereotypical fatherhood and motherhood

During the 1970s several reforms aimed at making it easier for both sexes to combine family and work were established in Sweden. The reforms pertinent to the present study were the introduction of a parental insurance system in 1974 which enabled fathers to take paid paternity leave, and in 1979 both parents became entitled to decrease their working hours until their child was 8 years old.

Paternity leave was selected as indicator of a less gender-stereotypical parental position for men. Information on full days of parental leave allowance during 1978–1979 was derived from the Social Insurance Register (National Social Insurance Board). At that time the entitlement was 270 days per child (i.e. 240 days with 90% of the individual income and 30 days with 32 Swedish crowns per day, about 10 Euros at present). Parents were entitled to utilise their parental leave insurance until their child was 8 years. For women, the selected indicator of a less gender-stereotypical motherhood was full-time work in 1980. The information on working hours came from the Swedish Population and Housing Censuses (Statistics Sweden) in which the respondents were asked to report their circumstances during the week 8–14 April 1980. The main reasons for using different periods for the male (1978–1979) and female (1980) exposures were: 1) most parental leave days are utilised during the child's first two years, 2) the mothers should be able in principle to choose between part-time or full-time work which was more likely in 1980 (when the average age of the children in the cohort was two years) than in 1978–79 (when the children were less than two years), and 3) census data is only available every 5 years.

### Confounders

The following variables were evaluated for confounding: age (continuous variable: years); country of birth (dichotomous variable: Sweden or other country); municipality 1980 (279 categories); income 1980 (continuous variable: Swedish crowns); 8 occupational positions 1980 (categories ranked by dominance: no independent occupational position, unskilled manual workers, routine non-manuals, skilled manual workers, routine non-manuals, intermediate non-manuals, self-employees and farmers, and higher managers and professionals). In the male analysis, we also considered type of work in 1980 (9 categories: administration; commerce; farming-, forest- and fishery; mining and stone; transport- and communication; manufacturing; service; military; science-, technical-, social-, humanistic- and artistic work). It should be noted that occupational position is derived from a combination of occupation itself and the educational level required for that occupation, which in part compensated for the fact that data on educational attainment were missing. Both the individual's choice of a caring versus working position, and his/her alcohol consumption, may also be affected by the life situation of his/her partner. Hence, the investigation of confounding also included the partner's age, country of birth, income in 1980, occupational position in 1980, female type of work (in the male analysis), maternity leave during 1978–1979 (in the male analysis), male working hours in 1980 (in the female analysis), and the partner's alcohol-related care/death during 1981–2001.

The potential confounding variables were obtained from the Total Population Register (age and municipality), the Migration Register (country of birth), the Income and Assets Register (income), and the Swedish Population and Housing Censuses (occupational position, type of work, and working hours based on circumstances during the week 8–14 April 1980), Statistics Sweden; the Social Insurance Register, National Social Insurance Board (parental leave for female partners); the Hospital Discharge and Cause of Death Registers, Swedish National Board of Health and Welfare (the partner's alcohol-related care and/or death).

### Statistical analysis

We fitted multivariate logistic regression models with odds ratios used as estimates of the relative risks. The main analyses were performed with dichotomized independent variables: paternity leave (1 day or more) versus no paternity leave (0 days); and full-time work (more than 34 hours per week) versus no gainful employment or part-time work (34 hours or less per week). We also performed analyses using multiple categories of paternity leave and working hours. In the male analysis, the risks of alcohol-related care/death among fathers with 1–30 days, 31–90 days, and more than 90 days were compared with the risk among fathers with 0 days of paternity leave. For females, the risks of alcohol-related care/death among mothers working 20–34 hours and more than 34 hours were compared with the risk among mothers not gainfully employed or working 19 hours or less per week. In order to check the consistency of results, the main analysis was also separately performed for alcohol-related inpatient care and alcohol-related mortality.

The final analyses included basic adjustments for age and municipality (Model I), plus socioeconomic position (Model II), plus the partner's age and socioeconomic position (Model III), plus the partner's exposure (parental leave or working hours) and the partner's alcohol-related care/death (Model IV). The results from Model IV was analysed by strata and tested for interaction (p-values for the product term); the likely effect-modifiers assessed were age (quartiles), occupational position ('manual workers', 'non-manual workers', 'miscellaneous occupational positions' including self-employment, farmers, retired, homemakers, and unclassified workers), cohabiting in 1980 (yes/no), sharing parenthood with a woman who worked full-time or not (for men), and sharing parenthood with a man who took paternity leave or not (for females). The potential public health impact was assessed by calculating the etiologic fraction: p × ((OR-1)/OR), where p is the proportion of exposed individuals (paternity leave and full-time work) among total cases with alcohol-related care and/or death (males and females respectively), and OR refers to the odds ratios (Model IV) for the exposed versus not exposed [30].

All analyses were performed in SPSS. Statistical significance was set at p = 0.05 and 95% confidence intervals (CI) were also calculated. The study was approved by the Research Ethics Committee at Umeå University during April 2004 (§ 178/03, 03-112).

## Results

The description of the original sample of fathers (n = 49,120) by category of paternity leave (Table 1) shows that not taking paternity leave was by far the most common strategy (39,136 fathers). Men who took 31–90 days earned on average most, were most likely to have more than one child, and had the highest proportion of medium/high non-manual jobs. These fathers also had the lowest death rates, days of inpatient care, and alcohol-related care/death. Fathers who took more than 90 days were worst off with respect to income, while fathers who took 1–30 days had the highest share of manual/low non-manual jobs. The highest risk of alcohol care/death was found among fathers with 0 days of paternity leave.

**Table 1:** Characteristics of the fathers in the original population by paternity leave days taken in 1978–1979: numbers, means, and proportions (%).

	Paternity leave 0 days	Paternity leave 1–30 days	Paternity leave 31–90 days	Paternity leave > 90 days
Number (n = 49,120)	39,136	5,351	3,396	1,237
Mean year of birth	1949	1949	1948	1948
Mean income 1980	70,288 SEK	74,011 SEK	75,854 SEK	68,893 SEK
Children in 2000:				
- one child	34.2%	31.8%	27.1%	33.4%
- two children	41.3%	44.2%	46.6%	40.9%
- three or more children	24.5%	24.0%	26.3%	25.7%
Born in Sweden	79.3%	89.0%	90.7%	87.7%
Cohabitation 1980	85.4%	92.3%	92.6%	90.4%
Occupational position 1980:				
- manual/low non-manual	46.7%	50.58%	40.0%	37.2%
- medium/high non-manual	21.3%	31.3%	42.1%	39.0%
- self-employed	8.3%	4.5%	3.9%	5.7%
- other	23.7%	13.6%	14.0%	18.0%
Deaths 1981–2001	4.6%	4.0%	3.2%	5.3%
Inpatient care 1981–2001	16 days	13 days	12 days	17 days
Alcohol-related inpatient care and/or death 1981–2001	4.7%	3.8%	3.4%	3.7%

Among the original female population (n = 43,450, Table 2), working 20–34 hours was the most common scenario (18,492 mothers). This category also had the lowest numbers of inpatient care days, and the lowest proportions of deaths and of alcohol-related care/death, along with the highest proportion of Swedish-born women. Full-time work was associated with the highest income and working 0–19 hours per week with the lowest. Mothers working more than 34 hours per week were also most likely to have a manual as well as a medium/high non-manual occupation. Finally, cohabiting in 1980 was most common among mothers who reported working 0–19 hours per week.

**Table 2:** Characteristics of the mothers in the original population by working hours per week in 1980: numbers, means, and proportions (%).

	Working hours 0–19	Working hours 20–34	Working hours > 34
Number (n = 43,450)	14,616	18,492	10,342
Mean year of birth	1952	1951	1951
Mean income 1980	20,317 SEK	42,459 SEK	55,864 SEK
Children in 2000:			
- one child	29.1%	32.6%	38.9%
- two children	41.8%	46.5%	39.6%
- three or more children	29.1%	20.9%	21.5%
Born in Sweden	86.0%	92.2%	83.8%
Cohabitation 1980	90.4%	89.0%	83.1%
Occupational position 1980:			
- manual	7.1%	42.5%	46.9%
- low non-manual	1.8%	29.0%	20.9%
- medium/high non-manual	3.1%	26.1%	26.7%
- self-employed	0.4%	1.9%	4.5%
- other	87.6%	0.5%	1.0%
Deaths 1981–2001	2.5%	1.7%	2.1%
Inpatient care 1981–2001	28 days	19 days	22 days
Alcohol-related inpatient care and/or death 1981–2001	2.4%	1.1%	2.1%

An analysis of individuals included in the regression analyses versus individuals excluded due to missing data on exposure or confounders, or due to having another child in 1979–1990, demonstrates statistically significant differences between the two groups (Table 3). The excluded fathers were less likely to take paternity leave, to work full-time, to be old, to have high income, to be born in Sweden, and to be cohabiting; besides, this group had a higher proportion of alcohol-related harm. The female picture is similar with two exceptions: excluded mothers were more likely to be cohabiting, and the proportions of alcohol-related harm were equal between individuals included and excluded from the regression analyses.

**Table 3:** Analyses of individuals (males and females) included in the regression analyses versus those excluded due to having another child in 1979–80 or missing data on covariates; proportions (%).

	Males in regression analyses	Males excluded due to missing	*p-values chi-square tests*	Females in regression analyses	Females excluded due to missing	*p-values chi-square tests*
	*n *= 25,150	*n *= 23,970		*n *= 33,406	*n *= 10,044	
Paternity leave 1978–79; > 0 days	23.1%	17.4%	< 0.001	-	-	-
Full-time work 1980, Mothers	-	-	-	25.2%	19.2%	< 0.001
Full-time work 1980, Fathers	93.6%	86.5%	< 0.001	-	-	-
Maternity leave 1978–79 = 0 day	-	-	-	98.8%	79.4%	< 0.001
Oldest quintile	21.4%	20.1%	= 0.001	24.4%	17.7%	< 0.001
Highest income quintile 1980	21.9%	17.5%	< 0.001	20.4%	18.8%	< 0.001
Born in Sweden	89.9%	72.3%	< 0.001	88.8%	66.8%	< 0.001
Cohabiting 1980	87.9%	85.8%	< 0.001	85.5%	90.7%	< 0.001
Alcohol-related inpatient care and/or death 1981–2001	3.9%	5.1%	< 0.001	1.8%	1.8%	0.998

Table 4 shows that men who took paternity leave have a consistent and statistically significant *lower *risk of alcohol-related inpatient care and/or death than men who did not take paternity leave; the crude results based on the original population demonstrate a decrease of 24%, the first Model (I) a decrease of 25%, and the final Model (IV) a decrease of 18%. The categorized analyses suggest that paternity leave beyond 90 days could be associated with the strongest decrease in risk of alcohol harm.

**Table 4:** Odds ratios of alcohol-related inpatient care and/or death among fathers for paternity leave (> 0 days) versus no paternity leave (0 days), and for three categories of paternity leave (1–30, 31–90, > 90 days) versus no paternity leave (0 days); crude (original population and population without those having another child in 1979–80) and models I-IV (95% CI).

	Odds ratios Crude	Odds ratios Crude	Odds ratios Model I	Odds ratios Model II	Odds ratios Model III	Odds ratios Model IV
	Original male population	Excluded: child 1979–80				
	*n *= 49,120	*n *= 38,031	*n *= 25,150	*n *= 25,150	*n *= 25,150	*n *= 25,150
***Dichotomized:***						
Paternity leave = 0 days *n = 19,341; alcohol harm = 799*	1	1	1	1	1	1
Paternity leave > 0 days *n = 5,809; alcohol harm = 182*	0.76 (0.68–0.86)	0.75 (0.66-0.87)	0.75 (0.64–0.89)	0.80 (0.68–0.95)	0.82 (0.69–0.99)	0.82 (0.68–0.99)

***Categorized:***						
Paternity leave = 0 days *n = 19,341; alcohol harm = 799*	1	1	1	1	1	1
Paternity leave: 1–30 days *n = 3,114; alcohol harm = 99*	0.79 (0.68–0.92)	0.78 (0.66–0.92)	0.77 (0.62–0.95)	0.80 (0.65–1.00)	0.82 (0.66–1.02)	0.82 (0.66–1.02)
Paternity leave: 31–90 days *n = 2,013; alcohol harm = 63*	0.71 (0.58–0.86)	0.71 (0.57–0.88)	0.75 (0.58–0.97)	0.82 (0.63–1.07)	0.85 (0.64–1.13)	0.85 (0.64–1.13)
Paternity leave: > 90 days *n = 682; alcohol harm = 20*	0.78 (0.58–1.06)	0.76 (0.54–1.06)	0.69 (0.44–1.09)	0.73 (0.46–1.15)	0.74 (0.45–1.23)	0.74 (0.45–1.2)

Model I: adjustments for age, country of birth, and municipality (missing: 2,381)

Model II: I + adjustments for income, occupational position, and type of work (missing: 5,626)

Model III: II + adjustments for partner's age, country of birth, income, occupational position, type of work, and parental leave (missing: 12,881)

Model IV: III + adjustments for partner's exposure (working hours) and the partner's outcome (alcohol-related care and/or death) (missing:12.881)

Table 5 shows that mothers who worked full-time about two years after having the child have a statistically significant *higher *risk of alcohol-related inpatient care and/or death than women who were part-time workers (34 hours or less) or not gainfully employed. The crude analyses and Model I demonstrate increased risks of 24%, 30% and 26%. When adjusting for own socioeconomic position, the risk is further strengthened; Model II-IV report increased risks of 83%, 75% and 71%. In the categorical analyses, the crude results based on the original population and on those who did not have another child in 1979–80, and the result in Model I, demonstrate that mothers who worked 'long' part-time (20–34 hours) had lower risks of alcohol harm (53%, 55% and 54%) than mothers not working or working 'short' part-time (0–19 hours). The socioeconomic adjustments (Model II) alter these results; the decreased risk for 'long' part-time work is no longer statistically significant, and the indication of a decreased risk for full-time work becomes an indication of increased risk (neither association are statistically significant).

**Table 5:** Odds ratios of alcohol-related inpatient care and/or death among mothers for full-time work (> 34 hours) versus other categories (0–34 hours), and for working 20–34 hours and > 34 hours versus 0–19 hours; crude (original population and population without those having another child in 1979–80) and models I-IV (95% CI).

	Odds ratios Crude	Odds ratios Crude	Odds ratios Model I	Odds ratios Model II	Odds ratios Model III	Odds ratios Model IV
	Original female population	Excluded: child 1979–80				
	*n *= 43,450	*n *= 33,696	*n *= 33,406	*n *= 33,406	*n *= 33,406	*n *= 33,406
***Dichotomised:***						
Working hours: 0–34 hours *N = 24,995; alcohol harm = 425*	1	1	1	1	1	1
Working hours: > 34 hours, *n = 8,411; alcohol harm = 187*	1.24 (1.06–1.45)	1.30 (1.09–1.54)	1.26 (1.06–1.50)	1.83 (1.48–2.26)	1.75 (1.41–2.16)	1.71 (1.38–2.19)

***Categorised:***						
Working hours: 0–19 hours *N = 9,415; alcohol harm = 243*	1	1	1	1	1	1
Working hours: 20–34 hours *N = 15,580; alcohol harm = 182*	0.47 (0.40–0.56)	0.45 (0.37–0.54)	0.46 (0.38–0.55)	0.84 (0.55–1.27)	0.84 (0.55–1.28)	0.81 (0.53–1.23)
Working hours: > 34 hours *N = 8,411; alcohol harm = 187*	0.87 (0.73–1.03)	0.85 (0.70–1.03)	0.84 (0.69–1.02)	1.57 (1.02–2.39)	1.50 (0.98–2.29)	1.41 (0.92–2.17)

Model I: adjustments for age, country of birth, and municipality (missing: 0)

Model II: I + adjustments for income and occupational position (missing: 40)

Model III: II + adjustments for partner's age, country of birth, income, occupational position, and working hours (missing: 250)

Model IV: III + adjustments for partner's exposure (parental leave), and the partner's outcome (alcohol-related care/death) (missing: 0)

The separate analyses (not reported in any table) of alcohol-related mortality and alcohol-related inpatient care indicate similar risk patterns, although stronger results for mortality than for inpatient care. The odds ratios for mothers who worked full-time versus other mothers were 3.25 (1.46–7.20) regarding alcohol-related mortality and 1.68 (1.30–2.17) regarding alcohol-related care. Correspondingly, the odds ratios for fathers who took paternity leave versus other fathers were 0.63 (0.37–1.06) for mortality and 0.84 (0.69–1.02) for care.

The stratified analyses of the combined measure of alcohol-related mortality and/or inpatient care demonstrated few statistically significant odds ratios for fathers, and no statistically significant interaction were found for either of the sexes (not reported in any table). Yet, there are indications (though highly overlapping) of a stronger effect from paternity leave in the oldest quartile (0.74, CI: 0.53–1.04) versus the youngest quartile (0.90, CI: 0.60–1.35), and for fathers who shared parenthood with mothers working full-time (0.78, CI: 0.58–1.04) versus not working or working part-time (0.86, CI: 0.68–1.09). Moreover, the risk decrease appears to be eliminated for fathers not cohabiting in 1980 (1.11, CI: 0.76–1.61). As regards females, the results seem to be consistent across age groups and cohabitation status. However, the risk associated with full-time work was stronger for mothers who shared parenthood with men who took paternity leave (2.26, CI: 1.38–3.70) versus no paternity leave (1.62, CI: 1.27–2.05). For mothers in miscellaneous occupational positions (such as self-employed and farmers) the risk increase is non-significant (1.23, CI: 0.65–2.33).

The calculations of etiologic fraction showed that 14.7% (117 cases) of the alcohol-related cases of care/death among the fathers who did not take paternity leave would have been avoided if they took paternity leave ((0.8145 × ((1.22-1)/1.22)), where 1.22 = 1/0.82). Correspondingly, 12.7% (24 cases) of the alcohol harm among the mothers working full-time would have been avoided if they had been not gainfully employed or part-time workers (0.3056 × ((1.71-1)/1.71)).

## Discussion

### Main findings

This study has indicated that the risk of future alcohol-related care and/or death differs between parents who adopt a gender-stereotypical position in early parenthood and parents who adopt a less gender-stereotypical position. We found that fathers who took paternity leave in 1978–1979 had a 18% lower risk of alcohol harm in the following two decades than those who did not, while mothers who worked full-time close to having a child had 71% higher risk of alcohol harm than those who were unemployed or worked part-time. This supports our overall hypothesis that increased gender equality in the division of parental duties leads to gender convergence in alcohol consumption and alcohol harm via male risk decrease and female risk increase [14,29].

On the whole, individuals excluded from the regression analyses due to missing data on working hours and/or other confounders, or because they had another child within the next two years, had lower socioeconomic status than individuals included; for males, the level of alcohol harm was also higher. Additionally, it was rarer that fathers in the excluded group took paternity leave and that mothers in the excluded group worked full-time. This could have distorted our findings; for example, the final male estimates could have been underestimated as the excluded fathers were both less likely to have the protective exposure of paternity leave and more likely to suffer alcohol harm. Yet, the male results are consistent from crude estimates based on the original population to the fully adjusted estimates, and the female analyses suggest that the adjustment for socioeconomic position (rather than the exclusion of individuals) had the strongest impact on the results. That is, when we control for the fact that mothers working full-time had higher income, and for the patterns of occupational position, the risk increase leaps from 26% to 83%.

As regarding the mothers, one should note that earlier research has mostly reported that paid work benefits women's health and well-being [10-12], which opposes our main finding. Yet, Båvner [15] found that women working more than 34 hours per week had twice the risk of sickness absence compared to other women, after controlling for earlier health and lifestyles. Furthermore, Burrows and Nettleton [18] reported that part-time work among women was associated with a 68% lower rate of risky alcohol behaviour than full-time work. A study that combined survey data and societal data also showed that, in countries with a strong welfare system like Sweden, women tended to drink more heavily if they had no formal education, if they were employed, and if they had a non-traditional family role [19].

The only statistically significant results in the categorical female analysis come from the crude and first model analyses, showing that 'long' part-time work associates with lower risks of alcohol harm than not working or working 'short' part-time. Yet, there is an indication of a U-shaped rather than linear relationship between an attenuated gender-stereotypical position and alcohol-related care and/or death, since working 0–19 hours or more than 34 hours appear to associate with higher risks than working 20–34 hours per week. This tentatively supports a combination of the theories of *expansion *and *stress *[9,10]; expanded roles may initially be protective through less opportunity and less need for risky drinking [15], but the stress experienced at the level of full-time work may ultimately increase the need for relaxation through alcohol use [8,13]. We found a statistically significant association between fathers who took paternity leave and mothers who worked full-time, i.e. the logical expectation that less gender-stereotypical parents are likely to share parenthood was supported. Perhaps, women who worked full-time were relieved from childcare duties but not from other stressful household duties. A reason for the potential benefits of 'long' part-time work versus the burden of full-time work could also be that the former is linked more regularly with picking up from daycares and the like, which does not fit well with risky drinking, while the latter strategy could in fact lead to more freedom to drink through more free time and money. This connects to the theory of *caring *according to which childcare duties per se are protective against risky behaviours [16]. But then, mothers not gainfully employed or loosely connected to the labour market in 1980 should have had the lowest risk of alcohol harm, which was not supported in our study. Potential reasons are that a considerable proportion in this category was stopped from 'long' part-time work and full-time work commitments because of risky drinking before motherhood (alcohol-related selection), or that 'long' part-time work is the best female position in terms of alcohol harm (although not necessarily in terms of power and resources).

The finding of lower risk of alcohol-related care/death from paternity leave supports the theory of *caring *(childcare per se prevents risky drinking) as well as the theory of *expansion *(more roles increase well-being/decrease the need for risky drinking). It should also be noted that the *stress *theory does not appear to apply to fathers, i.e. the extra childcare indicated by paternity leave was not so stressful as to lead to increased harmful alcohol consumption. There is a lack of studies to have investigated how various types of fatherhood affect health and longevity. Yet, a registry study on mortality caused by alcohol and narcotics among various groups of men [31] has shown that cohabiting fathers had the lowest death risk, that lone fathers with custody had a lower risk than childless cohabiting men, while lone fathers without custody of their children had the poorest outcomes. Altogether, this may be interpreted as an indication that a close and substantive parenting role may prevent health-damaging behaviours among men. Another study that concurs with our findings showed that parental leave among fathers (representing a caring position) was associated with 16% lower risk of all-cause mortality [16]. Finally, Bolzan, Gale and Dudley [20] have found that paternity leave could help decrease men's post-natal anxiety and stress, which are risk-factors for alcohol drinking.

### Causal direction

The main weakness is that data on risky alcohol consumption and on alcohol-related problems before the individuals became parents in 1978 was not available in our data set. Hence, the reported association between a less gender-stereotypical parenthood and alcohol-related care/death could be because they are both related to earlier drinking. Research on parental leave and health-related behaviours is generally lacking [32,33], as well as research on the causal direction between full-time work and risky alcohol drinking [18]. Therefore, one can only speculate about this potential selection bias. A father who habitually drinks alcohol to excess could have an incentive to take paternity leave instead of continuing paid work, which contradicts the explanation of reverse causation. At a certain level, however, the mother and others in his network are likely to recognise his drinking as problematic and attempt to stop the father from having the sole responsibility of caring for the child. Similarly, mothers who habitually drink alcohol to excess may have an incentive to enter full-time work to gain the resources and the freedom to drink, which supports the explanation of reverse causation. At a certain point, however, she is likely to abandon work in order to hide or avoid dealing with a problematic drinking. Although we were unable to adjust for alcohol-related factors before parenthood, we have adjusted for several factors associated with frequency of parental leave, and with alcohol-related sickness, injuries and mortality [2,3,29,32,33]. Hence, confounding from earlier frequency and quantity of alcohol use will have to take place within rather confined social strata that are, in themselves, homogeneous with respect to alcohol consumption.

### Other methodological concerns

Regarding the exposures, we believe that paternity leave is the best available variable that can be derived from these registry data if one aims at examining fathers' adoption of duties traditionally linked to mothers [32]. A potential weakness is that the data on paternity leave is limited to two years (about 90% of the days granted) for the child born in 1978. Yet, it has been reported that the parental division of the remaining days are quite gender equal [34], and it has been argued that paternity leave during infancy has greater effects on the future division of unpaid duties and paid work [33], and on sickness and mortality [16], than later paternity leave. Hence, we suggest that the male indicator of a less gender-stereotypical parenthood is valuable.

The corresponding indicator regarding a less gender-stereotypical motherhood is less obvious. One alternative indicator considered was 'decrease of maternity leave', but this was rejected on the grounds that any effects on alcohol harm are unlikely to be observed from, say, 270 to 240 days of leave. A problem with the choice of full-time work (in contrast to paternity leave) is that it correlates with working hours before motherhood [35]. Yet, full-time work about two years after having a child likely displays a more explicit occupational ambition or necessity than full-time work among women in general. Bernhardt [36] found that mothers who utilized their right to work part-time in 1980 would otherwise have chosen the "homemaker strategy". This supports our main analysis, where full-time work was assumed to indicate a breadwinner function and all other working positions chiefly a caring function.

The outcomes were focused on the long-term consequences of inpatient care and/or death due to alcohol psychosis, alcoholism, alcohol-related liver disease or alcohol intoxication. The exclusion of alcohol-related harm such as traffic accidents, drowning, falls, and intentional injuries [37] because of the lack of individually registered information is a weakness. Yet, a follow-up period of 21 years and the assumption of comparable gender and social patterns between included and excluded diagnoses/causes indicate that the findings are valid. The finding that alcohol-related care and alcohol-related death had similar risk patterns contributes also to strengthening our results.

Swedish health registers are generally deemed to be reliable and of high coverage [38]; for example, missing information on cause of death has been lower than 0.5% and on inpatient care lower than 1% during the studied period . Yet, the utilisation of registry data conveys potential faults due to missing data, misclassification, and cheating. An inspection by the National Social Insurance Board [39] showed that 8.3 percent of temporary childcare grants in 2004 were founded on incorrect grounds, which indicates that the paternity leave data could include some errors. Further, and as commented on repeatedly above, the missing cases regarding various labour market data from the Swedish Population and Housing Censuses in 1980 are problematic despite the high overall participation rate of 97.5%.

### Future research

The stratified analyses indicate that paternity leave has stronger protective effects on alcohol harm for old compared to young fathers. Hence, future studies could scrutinize how different motives for taking paternity leave associate with risky alcohol consumption; from a genuine ambition to take on concrete responsibility and care for their children, to unemployment or seeking leisure time. Further, the tendency that paternity leave appears to be most beneficial for fathers who share parenthood with mothers working full-time, and that the protective effect was eliminated for fathers who were not cohabiting with the child's mother, lead us to suggest further explorations based on the theories of expansion and stress among men; for example, could it be that the benefits from an expanded caring role (as indicated by taking paternity leave) are counteracted by burdens due to double-work and stress (as possibly indicated by the fact that the father is not living with the mother)? The utilisation of role theory in upcoming research is also motivated by the finding of a particularly strong risk of alcohol-related care and/or death from full-time work for mothers sharing parenthood with fathers who took paternity leave; could it be that these women have been forced or freer to adopt a breadwinner position, and risky drinking patterns, traditionally linked to men?

The above findings suggest that a parental position where the mother works full-time and the father takes paternity leave is most likely to be detrimental for women and beneficial for men in terms of alcohol harm. We believe that this potential conflict between increased gender equality among parents, and maternal healthiness and longevity, ought to be further explored. Based on the possible impact from occupational position on the relationship between full-time work and alcohol harm among mothers, it would also be interesting to know more about the possible different meanings of full-time work in terms of dedication to a professional career versus full-time work as economic necessity.

It is critical that future research considers drinking patterns and alcohol-related problems among mothers and fathers before they take on a gender-stereotypical parenting position, or not. Future studies could also usefully investigate the relationship between the gendered division of parental duties and risky alcohol drinking in terms of likely generation/cohort effects, which in our study is reflected in recently legislated formal opportunities to combine family and paid work for both sexes. Therefore, should an up-to-date parental population provide the same results?

## Conclusion

One implication of the gender system is that the meaning of childcare and paid work is gender-dependent. For example, men may receive praise for taking paternity leave, while women may be made to feel bad about working outside the family. Correspondingly, the reasons for and consequences of alcohol use differ between the sexes. Yet, the present study shows that fathers who adopt childcare duties have a lower risk of alcohol-related care and death, and that mothers who adopt full-time work close to having a child have a higher risk. We conclude that a less gender-stereotypical division of parental duties is likely to contribute to decreased gender disparity in alcohol-related sickness, injuries, and mortality.

## Competing interests

The authors declare that they have no competing interests.

## Authors' contributions

AM was project leader for the original design and collection of data, and led the writing of the manuscript. MB had the main responsibility for the alcohol-related information, and JH was senior advisor regarding the epidemiological methodology. All authors discussed the theoretical background and interpreted the findings. All authors read and approved the final manuscript.

## Pre-publication history

The pre-publication history for this paper can be accessed here:


